# Effect of Ensiling Density and Storage Temperature on Fermentation Quality, Bacterial Community, and Nitrate Concentration of Sorghum-Sudangrass Silage

**DOI:** 10.3389/fmicb.2022.828320

**Published:** 2022-02-18

**Authors:** Chunsheng Bai, Gang Pan, Ruoxuan Leng, Wenhua Ni, Jiyun Yang, Juanjuan Sun, Zhu Yu, Zhigang Liu, Yanlin Xue

**Affiliations:** ^1^College of Horticulture, Shenyang Agricultural University, Shenyang, China; ^2^Institute of Grassland Research, Chinese Academy of Agricultural Sciences, Hohhot, China; ^3^College of Grassland Science and Technology, China Agricultural University, Beijing, China; ^4^Inner Mongolia Sihai Agriculture and Animal Husbandry Technology Co., Ltd., Baochang, China; ^5^Inner Mongolia Engineering Research Center of Development and Utilization of Microbial Resources in Silage, Inner Mongolia Academy of Agriculture and Animal Husbandry Science, Hohhot, China; ^6^Inner Mongolia Key Laboratory of Microbial Ecology of Silage, Inner Mongolia Academy of Agriculture and Animal Husbandry Science, Hohhot, China

**Keywords:** sorghum-sudangrass silage, storage temperature, ensiling density, fermentation quality, bacteria community, nitrate

## Abstract

This study aimed to evaluate the fermentation quality, bacterial community, and nitrate content of sorghum-sudangrass silage with two ensiling densities [550 kg fresh weight (FW)/m^3^ (low density, LD) and 650 kg FW/m^3^ (high density, HD)] stored at two temperatures [10°C (low temperature, LT) and 25°C (normal temperature, NT)] for 60 days. The fermentation parameters, microbial counts, bacterial community, nutritional composition, and nitrate and nitrite levels were assessed. The pH and ammonia nitrogen (N) in all silages were below 4.0 and 80 g/kg total N, respectively. Compared with LT treatments, NT treatments had lower pH and lactic acid (LA) bacteria and yeasts counts and contained higher LA and LA/acetic acid (LA/AA) (*p* < 0.05). The LT-LD contained more ammonia–N than LT-HD (*p* < 0.05) and had higher nitrate and lower nitrate degradation than other treatments (*p* < 0.05). *Lactobacillus* was the most dominant genus with all treatments (57.2–66.9%). The LA, LA/AA, and abundances of *Pantoea*, *Pseudomonas*, and *Enterobacter* in the silage negatively correlated with nitrate concentration and positively correlated with nitrate degradation (*p* < 0.05). Moreover, pH and ammonia–N were positively correlated with nitrate concentration and negatively correlated with nitrate degradation (*p* < 0.05). Overall, all silage had satisfactory fermentation quality, and the silage with HD and NT had better fermentation quality and higher nitrate degradation. The bacterial communities in all silages were dominated by *Lactobacillus*. The nitrate degradation during the fermentation process might be related to the fermentation quality and the activity of *Pantoea*, *Pseudomonas*, and *Enterobacter* in silage.

## Introduction

Sorghum-sudangrass is widely cultivated as a roughage for ruminants in arid and semiarid areas, because of its strong drought resistance, high water-use efficiency, and satisfactory biomass production (Han et al., 2015; McCary et al., 2020). Previous studies revealed that sorghum-sudangrass silage generally has high fermentation quality, that ensiling it with chemical additives improves *in vitro* digestibility and aerobic security, and that the inoculation of lactic acid (LA) bacteria (LAB) promotes fermentation and reduces yeasts count (Han et al., 2015; Diepersloot et al., 2021). Replacing 35 or 45% of whole-plant corn silage with sorghum-sudangrass silage in dietary dry matter (DM) improves dairy cow performance and optimizes the rumen environment (Dann et al., 2008).

Storage temperature and ensiling density are the main environmental factors that influence the fermentation quality, aerobic stability, and microbial community of silage (Weiss et al., 2016; Brüning et al., 2018; Sun et al., 2021a). Silage stored at high temperature (from 35 to 41°C) contains lower organic acid concentrations than those stored at 20 to 30°C (Weinberg et al., 1998; Kim and Adesogan, 2006; Weiss et al., 2016). Furthermore, a high packing density can positively affect the fermentation quality and aerobic stability of maize silage (Jungbluth et al., 2017; Brüning et al., 2018). Guan et al. (2020) revealed that *Lactobacillus* dominated the fermentation process in whole-plant corn silage stored at 30°C, but *Lactobacillus* exhibited decreasing abundance after 3 days of ensiling when the silage was stored at 45°C. Zhang et al. (2018) also reported that LAB genera were present as main taxa in alfalfa silage stored at 20 and 30°C but were minor taxa in silage stored at 40°C. Moreover, Sun et al. (2021a) found that increasing the ensiling density can increase the abundance of *Lactobacillus* in barley silage. However, no study has yet addressed the effect of temperature and density on the fermentation quality and microbial community of sorghum-sudangrass silage.

Nitrate accumulation in sorghum-sudangrass is a serious problem for livestock and human health; because there is high potential to accumulate nitrate in the background of large amount of nitrogen fertilizer application (Zhang et al., 1999; Harada et al., 2000). This also limits the application range of sorghum-sudangrass as animal feed. The nitrate content in plants is mainly associated with nitrogen fertilization, weather factors, and the growth stage (Drewnoski et al., 2019; Holman et al., 2019; Rashid et al., 2019). However, nitrate is not always toxic to animals and can cause toxicity when its concentration is greater than 3,000 mg/kg in forage (Roozeboom et al., 2019). Ensiling is adopted as an effective method to degrade nitrate in forage and to preserve forage based on anaerobic microbial fermentation (Khorasani et al., 1997). During fermentation, plant nitrate reductase, enterobacteria, and lactobacilli degrade nitrate to generate nitrite and nitric oxide as intermediates and then to ammonia and nitrous oxide as end-products (Spoelstra, 1985). To our knowledge, nitrate, nitrate degradation and the correlation between nitrate and fermentation quality or bacterial community have not been characterized in sorghum-sudangrass silage to date.

When harvesting sorghum-sudangrass in September in Shenyang, China, the temperature ranges from 10 to 25°C, and it is challenging to prepare silage with good fermentation quality. With reference to the typical temperatures during ensiling period in northeast China, sorghum-sudangrass was ensiled with two ensiling densities [550 and 650 kg fresh weight (FW)/m^3^] and two storage temperatures (10 and 25°C). It was hypothesized that the differences in ensiling density and storage temperature will change the fermentation quality, microbial community, and nitrate content of sorghum-sudangrass silage. Thus, the sorghum-sudangrass silages prepared with two ensiling densities and stored at two ambient temperatures were analyzed for the fermentation parameters, microbial counts, bacterial community, nutrient composition, nitrate, and nitrite.

## Materials and Methods

### Silage Preparation

Sorghum-sudangrass (*Sorghum bicolor* × *sudanense*, variety, BMR Octane) was planted on an experimental farm of Shenyang Agricultural University (123°25′E, 41°46′N, mean annual temperature of 6.5°C, annual precipitation of 700 mm) and was harvested at the flag leaf ligule of the stem elongation stage on 28 September 2018, from three fields as repeticates. The forage from each field was separately chopped to a length of 1–2 cm using a forage chopper (ZT0.8, Xinnong Machinery Co. Ltd., Donggang, China). After mixing homogeneously, the chopped forage from each field was randomly divided into four batches. Two batches were placed into two plastic silos (diameter × height, 20 × 30 cm) by hand with sterile gloves at a density of 550 kg FW/m^3^ (low density, LD), respectively; the other two batches were also put into the two same siloes at a density of 650 kg FW/m^3^ (high density, HD). After packing, the silo was closed using a plastic cover with a rubber O-ring and screw. After sealing, the two silos with the same density from each field were stored for 60 days in constant temperature incubators at 10°C (low temperature, LT) and 25°C (normal temperature, NT).

### Fermentation Quality and Nutrient Composition

Silage silos were opened after 60 days of ensiling. A mixture of 20 g of silage and 180 ml of distilled water was homogenized for 100 s in a flap-type sterile homogenizer (JX-05, Shanghai Jingxin Industrial Development Co. Ltd., Shanghai, China) and filtered through four layers of cheesecloth to prepare silage extracts (Sun et al., 2021b). A pH meter with a glass electrode (PB-10, Sartorius Group, Göttingen, Germany) was used to detect the pH of the silage. Phenol-sodium hypochlorite colorimetry was used to detect the ammonia–N content (Broderick and Kang, 1980). The concentrations of LA, acetic acid (AA), propanoic acid (PA), and butyric acid (BA) in silage were assessed using a high-performance liquid chromatography (HPLC; Waters1525, Waters Corporation, Milford, MA, United States). The analysis conditions were as follows: chromatographic column, Carbomix H-NP5 (Sepax Technologies, Inc., Newark, DE, United States); detector, refractive index detector (Waters2414, Waters Corporation, Milford, MA, United States); mobile phase, 2.5 mmol/L H_2_SO_4_; flow rate, 0.6 ml/min; column temperature, 55°C; sample volume, 10 μl (Nazar et al., 2021). The organic acid concentrations were obtained by comparing the curves of the silage extracts with the standard curve of standard substances.

The fresh material or silage was dried at 65°C for 48 h, followed by drying at 105°C until a constant weight was achieved. The DM content of the silage was corrected for the loss of volatiles during drying according to Weissbach and Strubelt (2008). Dried samples were passed through a 1-mm screen for further chemical analysis. Total nitrogen (TN) was measured by the Kjeldahl method using an autoanalyzer (Kjeltec 8400; FOSS Co. Ltd., Hillerød, Denmark) with copper sulfate as a catalyst (AOAC, 1990) and the crude protein (CP) concentration was calculated by multiplying the TN concentration by 6.25. The concentrations of neutral detergent fiber (NDF) and acid detergent fiber (ADF) were measured according to the Van Soest method (Van Soest et al., 1991) using an Ankom 2000 fiber analyzer (Ankom, Macedon, NY, United States), without using a heat stable amylase, and were expressed inclusive of ash. Water-soluble carbohydrates (WSCs) were determined using anthrone colorimetry (Thomas, 1977).

### Microbial Counts and Bacterial Community

Fresh forage or silage (10 g) was placed into 90 ml of sterile distilled water and shaken at 4°C at 180 rpm for 30 min. Then, it was diluted successively to 10^–1^ to 10^–6^ to determine microbial counts according to Wang et al. (2021). The LAB, coliform bacteria, and yeast (molds) were enumerated on Man Rogosa Sharpe agar, Violet Red Bile agar, and Rose Bengal agar (Beijing Aoboxing Bio-tech Co. Ltd., Beijing, China), respectively, as reported by Cai et al. (1999).

Each silage sample (10 g) was placed into 90 ml of sterile distilled water and shaken using a cryogenic oscillator (THZ-98C, Shanghai Yiheng Scientific Instrument Co. Ltd., Shanghai, China) at 4°C and 180 rpm for 30 min. Then, it was filtered with a piece of four-layer gauze to obtain the filtrate, which was then centrifuged using a refrigerated centrifuge (ST 16R, Thermo Fisher Scientific, Inc., Waltham, MA, United States) at 4°C and 8,000 rpm for 15 min. Sediments enriched with bacteria were finally obtained for high-throughput sequencing (Wang et al., 2018). Total DNA of the bacterial community was extracted according to the instructions of the E.Z.N.A.^®^ soil DNA kit (Omega Bio-tek, Norcross, GA, United States). A bead-beating step (Precellys^®^ 24 tissue homogenizer, Bertin Instruments, Montigny-le-Bretonneux, France) was used to increase DNA recovery. The quality of DNA was assessed using spectrophotometry (ND 8000, NanoDrop^®^ Technologies, Thermo Scientific, Waltham, MA, United States). Then, the variable region of V3–V4 of 16S ribosomal RNA (rRNA) was amplified with the primers 338F (5′-ACTCCTACGGGAGGCAGCAG-3′) and 806R (5′-GGACTACHVGGGTWTCTAAT-3′) (Sun et al., 2021a). The PCR reaction system included the following: 5× TransStartFastPfu buffer solution, 4 μl; 2.5 mM dNTPs, 2 μl; upstream primer (5 μM) and downstream primer (5 μM), 0.8 μl each; TransStart FastPfu DNA polymerase, 0.4 μl; template DNA, 10 ng; all supplemented with inactivated deionized water to 20 μl. The AxyPrep DNA Gel Extraction Kit (Axygen Biosciences, Union City, CA, United States) was used to purify the PCR products, and a Quantus™ Fluorometer (Promega, United States) was used for quantitative determination, using the MiSeq PE300 platform of the Illumina Company for sequencing (Shanghai Majorbio Bio-Pharm Technology Co. Ltd.). Next fastp^1^ and FLASH^2^ software were used to control quality and splice raw sequences, respectively. Furthermore, UPARSE software^3^ was used to cluster sequences with 97% similarity in operational taxonomic units (OTUs). Then, RDP classifier^4^ and Silva 16S rRNA database (v138) were used to analyze the microbial composition. Principal coordinates analysis of the abundance of bacterial communities (at genus level) in silage and the difference in bacterial communities (genus level) in silage was performed by R (version 3.2.1). The sequence data were deposited in the NCBI Sequence Read Archive under the accession number SRP272464.

### Nitrate and Nitrite Concentrations

For this, 5 g of each dried sample was extracted with 250 ml of phosphate buffer (Na_2_HPO_4_⋅12H_2_O, 1.79 g; NaH_2_PO_4_⋅2H_2_O, 0.78 g; NaClO_4_, 14.04 g; plus distilled water for a constant volume of 1 L) and then shaken at 180 rpm for 20 min and filtered. The nitrate and nitrite concentrations were assessed using HPLC (Waters1525, Waters Corporation, Milford, MA, United States). The analysis conditions according to the manufacturer’s instructions for the chromatographic column were as follows: chromatographic column, NH2P-50 4E (Shimadzu Corporation, Tokyo, Japan); detector, UV detector (Waters2489, Waters Corporation, Milford, MA, United States); wavelength, 210 nm; mobile phase, phosphate buffer (identical with the extract); flow rate, 0.8 ml/min; column temperature, 40°C; sample volume, 10 μl. The nitrate degradation rate was calculated as follows: (nitrate in fresh materials − nitrate in silage) / nitrate in fresh materials × 100.

### Statistical Analysis

Microbial counts were log-transformed before the statistical analysis. Data on fermentation quality, microbial counts, nutrient composition, nitrate, and nitrite were analyzed using a 2 × 2 factorial design. The model included two storage temperatures, two ensiling densities, and their interactions. A general linear model of statistical software SPSS (23.0) was used to perform two-way ANOVA for temperatures and densities, together with their interaction. One-way ANOVA was used to statistically analyze the difference among the treatments (*p* < 0.05). Meanwhile, the correlations between the nitrate concentration and nitrate degradation rate with fermentation parameters (pH, LA, AA, LA/AA, and ammonia–N) or main bacterial genera (top 10 most abundant genera) were analyzed by R (version 3.2.1) at https://www.omicstudio.cn/tool/59.

## Results

### Characteristics of Fresh Forage

The concentrations of CP, NDF, ADF, and WSC in sorghum-sudangrass before ensiling were 68.9, 564, 334, and 168 g/kg DM, respectively (Table 1). The nitrate content was 2,688 mg/kg, and nitrite was no detected.

**TABLE 1:** Chemical composition of sorghum-sudangrass before ensiling (*n* = 3).

Items	Sorghum-sudangrass	SEM
DM (g/kg FW)	252	6.84
Crude protein (g/kg DM)	68.9	1.15
Neutral detergent fiber (g/kg DM)	564	4.85
Acid detergent fiber (g/kg DM)	334	7.35
Water soluble carbohydrates (g/kg DM)	168	5.29
Nitrate (mg/kg DM)	2,688	117.34
Nitrite (mg/kg DM)	ND	–

*SEM, standard error of the mean; ND, not detected.*

### Fermentation Quality and Nutrient Composition of Silage

The storage temperature mainly affected the pH, LA, and LA/AA of silage (*p* < 0.05), and the ensiling density mainly affected LA and ammonia–N (*p* < 0.05) (Table 2). Moreover, storage temperature and ensiling density interacted on ammonia–N (*p* < 0.05). The NT treatments had lower pH but higher LA and LA/AA than LT treatments (*p* < 0.05); moreover, the NT-HD contained greater LA than NT-LD (*p* < 0.05). The ammonia–N content in NT treatments was higher than that in LT-HD but lower than that in LT-LD (*p* < 0.05). The BA and PA contents were not detected with any of treatments (Table 2).

**TABLE 2:** Fermentation quality and nutrient composition of sorghum-sudangrass silage (*n* = 3).

Items		LT		NT		SEM	Significance		
		LD	HD	LD	HD		T	D	T × D
Fermentation quality	pH	3.99^a^	3.99^a^	3.55^b^	3.52^b^	0.07	**	NS	NS
	Lactic acid (LA, g/kg DM)	28.3^c^	31.5^c^	63.1^b^	74.0^a^	6.06	**	*	NS
	Acetic acid (AA, g/kg DM)	20.1	17.8	19.7	22.3	0.93	NS	NS	NS
	Propionic acid (g/kg DM)	ND	ND	ND	ND	–	–	–	–
	Butyric acid (g/kg DM)	ND	ND	ND	ND	–	–	–	–
	LA/AA	1.41^b^	1.81^b^	3.21^a^	3.36^a^	0.26	**	NS	NS
	Ammonia–N (g/kg TN)	58.8^a^	33.7^c^	43.5^b^	43.6^b^	2.84	NS	*	**
Nutrient composition	Dry matter (g/kg FW)	245	262	274	280	10.5	NS	NS	NS
	Crude protein (g/kg DM)	64.6	66.0	60.5	62.7	1.11	NS	NS	NS
	Water soluble carbohydrates (g/kg DM)	67.6^a^	68.2^a^	24.0^c^	45.8^b^	5.79	**	*	*
	Neutral detergent fiber (g/kg DM)	568	582	574	590	4.00	NS	NS	NS
	Acid detergent fiber (g/kg DM)	366	371	362	378	6.37	NS	NS	NS
	Crude ash (g/kg DM)	73.9^ab^	75.2^a^	70.2^ab^	69.6^b^	0.85	*	NS	NS

*LT, storage temperature at 10°C; NT, storage temperature at 25°C; LD, ensiling density at 550 kg/m^3^; HD, ensiling density at 650 kg/m^3^.*

Means with different superscripts in the same row (a–c) differ significantly (p < 0.05). SEM, standard error of the mean. ND, not detected; *p < 0.05; **p < 0.01; NS, not significant; T, storage temperature; D, ensiling density; T × D, interactive effect of storage temperature and ensiling density.

The storage temperature mainly affected the crude ash concentration of the silage (*p* < 0.05), and the storage temperature and ensiling density had main effects and interactions on the WSC (*p* < 0.05) (Table 2). The LT treatments contained higher WSC than HD treatments (*p* < 0.05); moreover, the WSC in the NT-LD was lower than that in the NT-HD (*p* < 0.05).

### Microbial Counts and Bacterial Community in Silage

The storage temperature mainly affected the LAB and yeast counts of silage (*p* < 0.05), and the NT treatments had lower counts of LAB and yeast than the LT treatments *(p* < 0.05; Table 3). Molds were not detected with HD treatments; moreover, coliform bacteria were not detected with any of the treatments.

**TABLE 3:** Microbial counts, bacterial sequencing data, and alpha diversity of sorghum-sudangrass silage (*n* = 3).

Items		LT		NT		SEM	Significance		
		LD	HD	LD	HD		T	D	T × D
Microbial counts	Lactic acid bacteria (log_10_ cfu/g FM)	7.08^a^	6.76^a^	5.21^b^	5.47^b^	0.29	**	NS	NS
	Yeasts (log_10_ cfu/g FM)	6.34^a^	6.14^a^	3.98^b^	4.08^b^	0.38	**	NS	NS
	Molds (log_10_ cfu/g FM)	2.44	ND	2.33	ND	–	–	–	–
	Coliform bacteria (log_10_ cfu/g FM)	ND	ND	ND	ND	–	–	–	–
Sequencing data	Raw reads	46,103	45,334	52,155	46,816	1,947	NS	NS	NS
	Clean reads	42,211	40,532	50,720	45,251	2,065	NS	NS	NS
Alpha diversity	OTUs	84^b^	106^ab^	140^ab^	146^a^	11.5	*	NS	NS
	Shannon	1.593	1.908	1.919	1.933	0.085	NS	NS	NS
	Simpson	0.391	0.278	0.289	0.273	0.028	NS	NS	NS
	Ace	129^c^	204^bc^	320^a^	242^ab^	25.3	**	NS	*
	Chao 1	107^c^	157^bc^	234^a^	210^ab^	16.7	**	NS	NS
	Coverage	0.9995^a^	0.9991^ab^	0.9989^b^	0.9988^b^	0.0001	**	NS	NS

*LT, storage temperature at 10°C; NT, storage temperature at 25°C; LD, ensiling density at 550 kg/m^3^; HD, ensiling density at 650 kg/m^3^.*

Means with different superscripts in the same row (a–c) differ significantly (p < 0.05). SEM, standard error of the mean. ND, not detected; *p < 0.05; **p < 0.01; NS, not significant; T, storage temperature; D, ensiling density; T × D, interactive effect of storage temperature and ensiling density.

In total, 571,224 raw reads and 536,142 clean reads of the 16S rRNA genes were obtained from 12 silage samples according to sequencing data (Table 3). The average number of clean reads was 44,679 for each sample.

Storage temperature mainly affected OTUs, Ace and Chao 1 indexes, and coverage (*p* < 0.05); moreover, storage temperature and ensiling density interacted on Ace indexes (*p* < 0.05) (Table 3). The LT-LD had lower OTUs than the NT-TD (*p* < 0.05) and contained lower Ace and Chao 1 indexes but higher coverage than NT treatments (*p* < 0.05). Moreover, the NT-LD had higher Ace and Chao 1 indexes than the LT treatments (*p* < 0.05).

**FIGURE 1:**
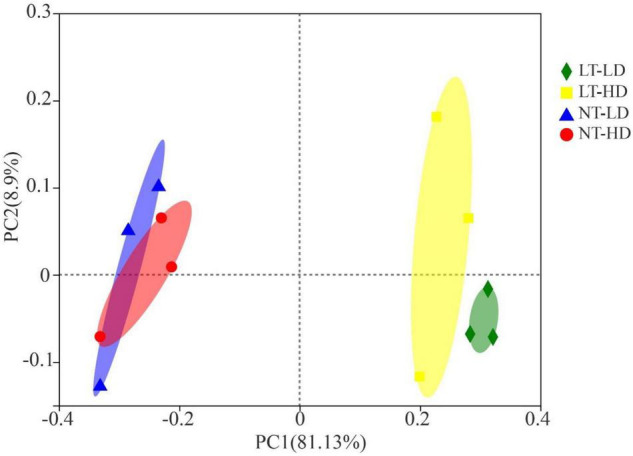
Principal coordinates analysis of the bacterial community for sorghum-sudangrass silage (*R*^2^ = 0.8362, *p* = 0.003; *n* = 3). LT, storage temperature at 10°C; NT, storage temperature at 25°C; LD, ensiling density at 550 kg/m^3^; HD, ensiling density at 650 kg/m^3^.

The NT treatments had clearly separated bacterial communities from LT treatments (Figure 1). The LT-LD and LT-HD had the separated bacterial communities; furthermore, the bacterial communities of NT-TD and NT-LD were clustered together.

**FIGURE 2:**
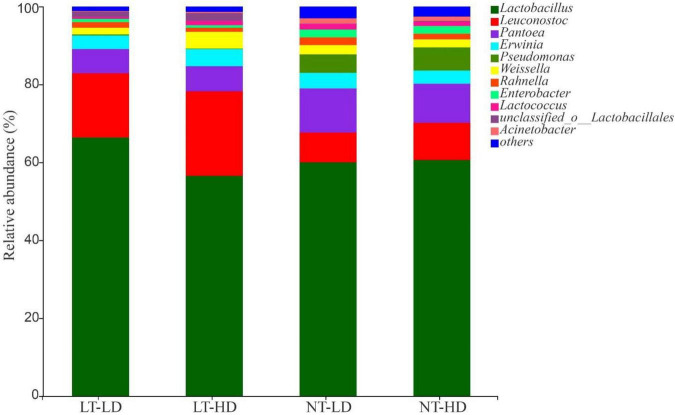
Bacterial community (genus level) of sorghum-sudangrass silage (*n* = 3). LT, storage temperature at 10°C; NT, storage temperature at 25°C; LD, ensiling density at 550 kg/m^3^; HD, ensiling density at 650 kg/m^3^.

**FIGURE 3:**
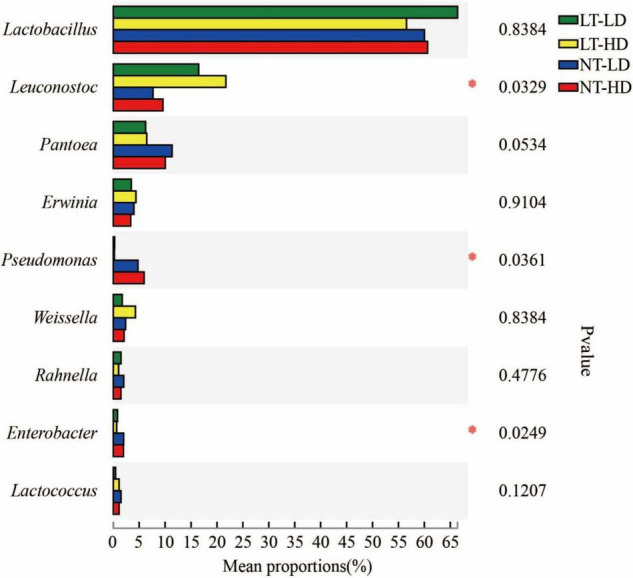
Difference in bacterial community (genus level) of sorghum-sudangrass silage (*n* = 3). LT, storage temperature at 10°C; NT, storage temperature at 25°C; LD, ensiling density at 550 kg/m^3^; HD, ensiling density at 650 kg/m^3^. *p < 0.05.

*Lactobacillus* was the most dominant genus in LT-LD, LT-HD, NT-LD, and NT-HD, with abundances of 66.85, 57.16, 60.16, and 60.70%, respectively (Figure 2). The other common main bacterial genera (>1% abundance) were *Leuconostoc*, *Pantoea*, *Erwinia*, *Weissella*, *Rahnella*, and unclassified *Lactobacillales* in LT treatments and *Leuconostoc*, *Pantoea*, *Erwinia*, *Pseudomonas*, *Weissella*, *Rahnella*, *Enterobacter*, *Lactococcus*, and *Acinetobacter* in NT treatments. Compared with LT treatments, NT treatments had lower *Leuconostoc* but higher *Pseudomonas* and *Enterobacter* (*p* < 0.05) (Figure 3).

### Nitrate and Nitrite Concentrations of Silage

Storage temperature and ensiling density had the main effects and interactions on the nitrate content and nitrate degradation rate of silage (*p* < 0.05; Table 4). The LT-LD contained more nitrate and lower nitrate degradation rate than other treatments (*p* < 0.05). Nitrite was not detected in any of the silage.

**TABLE 4:** Nitrate concentration, nitrate degradation rate, and nitrite concentration of sorghum-sudangrass silage (*n* = 3).

Items	LT		NT		SEM	Significance		
	LD	HD	LD	HD		T	D	T × D
Nitrate (mg/kg DM)	1480^a^	868^b^	783^b^	645^b^	103	**	**	*
Nitrate degradation rate (%)	44.94^b^	67.72^a^	70.90^a^	76.02^a^	3.83	**	**	*
Nitrite (mg/kg DM)	ND	ND	ND	ND	–	–	–	–

*LT, storage temperature at 10°C; NT, storage temperature at 25°C; LD, ensiling density at 550 kg/m^3^; HD, ensiling density at 650 kg/m^3^.*

Means with different superscripts in the same row (a–c) differ significantly (p < 0.05). SEM, standard error of the mean. ND, not detected; *p < 0.05; **p < 0.01; T, storage temperature; D, ensiling density; T × D, interactive effect of storage temperature and ensiling density.

### Correlation Between Nitrate and the Fermentation Quality and Bacterial Community

**FIGURE 4:**
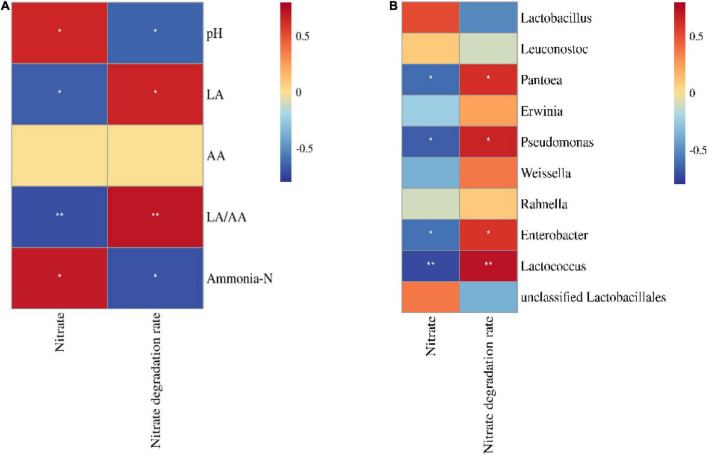
Correlation heatmap of nitrate and nitrate degradation rate with fermentation parameters **(A)** and the bacterial community (top 10 bacterial genera, **B**) (*n* = 12). LA, lactic acid; AA, acetic acid. *p < 0.05; **p < 0.01.

The nitrate concentration in silage was positively correlated with ammonia–N and pH (*p* < 0.05) and negatively correlated with *Pantoea*, *Pseudomonas*, *Enterobacter*, and *Lactococcus* (*p* < 0.05; Figures 4A,B). Moreover, the nitrate degradation rate was positively correlated with LA, LA/AA, *Pantoea*, *Pseudomonas*, *Enterobacter*, and *Lactococcus* (*p* < 0.05) and negatively correlated with pH and ammonia–N (*p* < 0.05).

## Discussion

### Material Characteristics and Silage Quality

In the present study, the fresh sorghum-sudangrass contained sufficient WSC (168 g/kg DM) as a fermentation substrate for microorganisms during the fermentation process, as reflected by the low pH and high LA content in NT treatments, with more than 24.0 g/kg of residual WSC (Tables 1, 2). These were in line with the results in the work of Han et al. (2015) and Diepersloot et al. (2021). Nevertheless, the fresh materials in the present study had lower DM contents than those in previous studies (252 vs. 254–256 and 349 g/kg) (Han et al., 2015; Diepersloot et al., 2021), because of the different harvesting stages (flag leaf ligule of stem elongation stage vs. after booting stage). Moreover, the nutrient compositions of sorghum-sudangrass at harvesting are also affected by the variety, irrigation levels, and nitrogen doses (Han et al., 2015; Kaplana et al., 2019).

According to the evaluating system for the fermentation quality of silage (Kaiser and Weiss, 2005), the scores with all treatments were 100, and their grade was first in the present study, owing to the lower contents of BA and AA in all silage (<3.0 and <30 g/kg DM, respectively). The normal storage temperature (25°C) might be a good temperature for higher LAB activity during the earlier fermentation process in silage, as reflected by the higher LA and lower pH in NT treatments (Table 2). Previous studies also revealed similar results regarding the effect of storage temperature on the fermentation characteristics of whole-plant corn silage (Zhou et al., 2016, 2019; Ferrero et al., 2021). However, the LAB in the final silage might be inhibited by the low pH condition (McDonald et al., 1991), which resulted in lower LAB count in NT treatments (Table 3). High ensiling density is favorable for reducing the porosity of forage granules in silos (Pitt and Muck, 1993; Toruk et al., 2009), emerging rapidly anaerobic environment, and mitigating WSC consumption during initial aerobic stage (Borreani et al., 2018). Moreover, in the present study, NT-HD contained greater LA than NT-LD (Table 2). These results indicated that HD contributes to the improvement in LAB fermentation in silage.

Lactic acid bacteria have lower activity in silage stored at LTs (Jungbluth et al., 2017), which might cause LT treatments contained lower organic acid concentrations than NT treatments. Nevertheless, the bacterial community in NT and LT treatments was absolutely dominated by homofermentative LAB (*Lactobacillus plantarum*) and heterofermentative LAB (*Lactobacillus brevis*), respectively (Supplementary Figure 1), and the LT treatments had more *Leuconostoc*, as heterofermentative LAB (Figure 3). Homofermentative LAB produce only LA, whereas heterofermentative LAB produce LA, AA, alcohols, and esters (Kung et al., 2018). These results suggest that, in the present study, the synergy of storage temperature and microbial dominant bacterial species resulted in higher LA and LA/AA in NT treatments than in LT treatments, with no difference in AA among all treatments (Table 2). Ammonia–N as a non-protein in silage indicates silage preservation during fermentation (Thomas et al., 1980). In the present study, all silages contained lower ammonia–N than the suggested contents (<60 g/kg TN vs. 80–120 g/kg TN) (Kung et al., 2018), because *Clostridium* activity is inhibited in silage with a pH below 4.2 (Driehuis et al., 2018). This was also reflected by the bacterial community (Figure 2). Moreover, Li et al. (2017) reported that the ammonia–N content in oat silage with 168.9 kg DM/m^3^ (507 kg FW/m^3^) of density increased as a decreasing storage temperature. Similarly, in the present study, LT-LD contained higher ammonia–N than NT-LD (Table 2). In the silage stored at low storage temperature, the microorganism has low activity, and the plant respiration is very low during initial aerobic phase; furthermore, there is relatively more air in silage with LD (McDonald et al., 1991; Toruk et al., 2009), and this silage has long the initial aerobic phase and slower fermentation rate. However, the proteolytic enzymes have a significant level of activity remaining under the condition of below 10°C and above pH 5.5 (Spoelstra, 1985). Those suggested that the low storage temperature might have negative effect on the ammonia–N content in silage with low ensiling density (≤500 kg FW/m^3^). Brüning et al. (2018) reported that, storing at 12°C, the maize silage with high ensiling density contained less ammonia–N content than that with LD; moreover, in the present study, the LT-HD had lower ammonia–N than LT-LD (Table 2). There was no difference in ammonia–N content between NT-LD and NT-HD in the present study (Table 2); this was in line to the results of maize and sorghum silages stored at 25–28°C (Sucu et al., 2016). Moreover, the LT-HD had lower ammonia–N than NT-HD in the present study (Table 2). Those abovementioned implied that rising ensiling density can decrease the ammonia–N content in final silage stored at low storage temperature, the ensiling density has no effect on ammonia–N content in final silage stored at suitable temperature for fermentation (25–30°C), and reducing storage temperature decreases ammonia–N content in final silage with high ensiling density.

In the present study, the storage temperature and ensiling density had the main effects and interactions on the WSC concentration in the final silage (Table 2). The NT treatments contained more LA but less WSC than LT treatments (Table 2). This indicated that the bacteria in silage stored at 25°C tended to be more vigorous and utilized more WSC during the fermentation process. The reason for this might be that the microorganisms have greater activity in silage stored at NTs (20–30°C) than at LTs (less than 10°C) (Zhou et al., 2016; Jungbluth et al., 2017). Compared with NT-HD, the NT-LD contained less LA and WSC, but there was no difference in AA content (Tables 2, 3), which showed that there might be more residual oxygen and active aerobes consumed more WSC at the early period of fermentation (Sucu et al., 2016; Anésio et al., 2017).

### Microbial Counts and Bacterial Community in Silage

In the present study, compared with LT treatments, the NT treatments contained lower counts of LAB and yeasts, lower pH, and higher LA content (Tables 2, 3). These results indicated that some microorganisms in the silage were inhibited during fermentation process under lower pH conditions (McDonald et al., 1991). This was also reflected by the correlation between the LAB and yeast counts and the pH and LA concentration (Supplementary Figure 2). Similar results were also reported in other studies (Ren et al., 2019; Wang et al., 2019a). Moreover, the LAB in LT treatments might develop tolerance of LT during fermentation process (60 days after ensiling), owing to specific conditions can contributed to the formation of the unique microorganisms (Pang et al., 2012). Molds were only detected in LD treatments, whereas mold count in HD treatments was below the detection limit (Table 3). The reason for this might be that the higher content of oxygen in silage with low ensiling density during the early ensiling period could lead to more mold residues (Tian et al., 2020).

The bacterial communities between LT and NT treatments were completely separated from each other. With low storage temperature, the communities in LD and HD treatments were adjacent to each other and clearly separated; however, the communities in NT-LD and NT-HD clustered together (Figure 2). These results indicated that storage temperature is the most important factor affecting the bacterial community, whereas ensiling density has less influence. Guan et al. (2020) also revealed that the storage temperature mainly affects the bacterial community of whole-plant corn silage. Zhou et al. (2016) and Wang et al. (2019a) further found that temperature treatment could cause the emergence of a separate bacterial community. These results indicated that storage temperature and ensiling density could induce the changes in the bacterial community structure of silage.

In the present study, *Lactobacillus* dominated the bacterial community of sorghum-sudangrass silage, with the highest abundance (Figure 2). Moreover, *Lactobacillus* is closely related to the LA content and pH of silage (Ni et al., 2018; Wang et al., 2019a) and is a characteristic of good fermentation (Guan et al., 2020). These results also explained the satisfactory fermentation quality of the silage in the present study. There was no difference in *Lactobacillus* in sorghum-sudangrass silage between high and LD (Figure 3); however, Sun et al. (2021a) reported that increasing density could enhance the abundance of *Lactobacillus* in barley silage. This difference might be due to differences in characteristics of forage before ensiling. Compared with NT treatments, the LT treatments contained more *Leuconostoc* and *L. brevis* as heterofermentative LAB (Figure 2 and Supplementary Figure 1), which showed that heterofermentative LAB might be highly resistant to LT. In addition, the LT treatments had lower LA/AA and higher pH than NT treatments (Table 2). These results suggested that heterofermentative LAB might dominate the fermentation process of sorghum-sudangrass silage under low storage temperature (Cai et al., 1998). In the present study, *Pantoea* was one of the main bacterial genera (6.18–11.35%) in sorghum-sudangrass silage; moreover, *Pantoea* exhibits increasing abundance as the most dominant bacterial genus during the initial aerobic phase of whole-plant corn silage (Sun et al., 2021a). However, the effect of *Pantoea* on silage is not clear and requires further study. The NT treatments had higher *Pseudomonas* than LT treatments in the present study (Figure 3); this genus was also detected in mulberry leaf silage and Moringa oleifera leaf silage with high abundance (Wang et al., 2019b,c). The silage subjected to high temperature contained more *Pseudomonas* than silage at lower temperature (Wang et al., 2019c). Those indicated that the emergence of *Pseudomonas* in silage is likely related to the variety of raw materials and is also affected by temperature. The optimum temperature for *Enterobacter* proliferation is quite high (Weiss et al., 2016), which was regarded as one of the reasons for its lower abundance at 10°C. Similar results were also reported by Zhou et al. (2019).

### Nitrate and Nitrite Concentrations of Silage

In the present study, fresh sorghum-sudangrass did not cause toxicity according to Roozeboom et al. (2019) (nitrate concentration <3,000 mg/kg). During fermentation, nitrite and nitric oxide, as intermediate products for nitrate degradation, can inhibit the growth of undesirable bacteria in the silage (Knicky and Sporndly, 2009), which explained the lack of *Clostridium* detected in silage in the present study (Figure 2). Nitrate begins to reduce immediately after ensiling, and nitrite and nitric oxide accumulate temporarily and disappear within 1 or 2 weeks (Spoelstra, 1985), as reflected by the lack of nitrite detected in silage in the present study (Table 4). The nitrate degradation rate in LT-LD was lower than that in LT-HD, but there was not difference between NT-LD and NT-HD (Table 4). Those indicated that high ensiling density improved nitrate degradation in the silage stored at LT but did not affect nitrate degradation at NT. Moreover, the nitrate degradation rate correlated positively with LA and LA/AA and positively with pH and ammonia–N (Figure 4A), suggesting that satisfactory fermentation quality contributed to nitrate degradation in silage. A previous study revealed that enterobacteria in silage are one of the main microbes that degrade nitrate during the fermentation process (Driehuis et al., 2018). In the present study, *Pantoea*, *Pseudomonas*, and *Enterobacter*, which are enterobacteria, were positively correlated with the nitrate degradation rate (Figure 4B); *Pseudomonas* and *Enterobacter* in NT treatments were more abundance than those in LT treatments (Figure 3). In addition, the optimal storage temperature for enterobacteria is higher than that for LAB (Weiss et al., 2016) and enterobacteria counts are greater in silage stored at 20°C compared with those at 10°C (Zhou et al., 2016). Those also explain that, in the present study, nitrate degradation was greater in NT treatments and influenced by the storage temperature (Table 3).

## Conclusion

Sorghum-sudangrass silage has satisfactory fermentation quality and is safe in terms of the nitrate concentration. The silage stored at 25°C and with a density of 650 kg/m^3^ had better fermentation quality and high nitrate degradation. *Lactobacillus* dominated the bacterial community, and storage temperature was the main factor affecting the bacterial community of the silage. The satisfactory fermentation quality contributed to the nitrate degradation of silage. *Pantoea*, *Pseudomonas*, and *Enterobacter* were the main bacterial genera degrading the nitrate in sorghum-sudangrass silage.

## Data Availability Statement

The original contributions presented in the study are included in the article/Supplementary Material, further inquiries can be directed to the corresponding author.

## Author Contributions

CB, JS, ZY, and YX designed the study and wrote the manuscript. CB, GP, RL, WN, JY, and ZL performed the experiments. JS, ZY, and YX reviewed and edited the manuscript. CB, GP, RL, and WN analyzed the data. ZY and YX funded and supervised the experiments. All authors reviewed the manuscript.

## Conflict of Interest

ZL is employed by Inner Mongolia Sihai Agriculture and Animal Husbandry Technology Co., Ltd. The remaining authors declare that the research was conducted in the absence of any commercial or financial relationships that could be construed as a potential conflict of interest.

## Publisher’s Note

All claims expressed in this article are solely those of the authors and do not necessarily represent those of their affiliated organizations, or those of the publisher, the editors and the reviewers. Any product that may be evaluated in this article, or claim that may be made by its manufacturer, is not guaranteed or endorsed by the publisher.

## Footnotes

1 https://github.com/OpenGene/fastp, version 0.20.0.

2 http://www.cbcb.umd.edu/software/flash, version 1.2.7.

3 http://drive5.com/uparse/, version 7.1

4 http://rdp.cme.msu.edu/, version 2.2

## Supplementary Material

Supplementary Figure 1 Bacteria community (species level) of sorghum-sudangrass silage (*n* = 3). LT, storage temperature at 10°C; NT, storage temperature at 25°C; LD, ensiling density at 550 kg/m^3^; HD, ensiling density at 650 kg/m^3^.

Supplementary Figure 2 Correlation networks among microbial counts and fermentation quality (*n* = 12). *p*-Value < 0.05.
