# Supplementary figures and images for: Effect of Ensiling Density and Storage Temperature on Fermentation Quality, Bacterial Community, and Nitrate Concentration of Sorghum-Sudangrass Silage
Source: Front Microbiol. 2022 Feb 18;13:828320. doi: 10.3389/fmicb.2022.828320 (PMC8895230; doi:10.3389/fmicb.2022.828320)

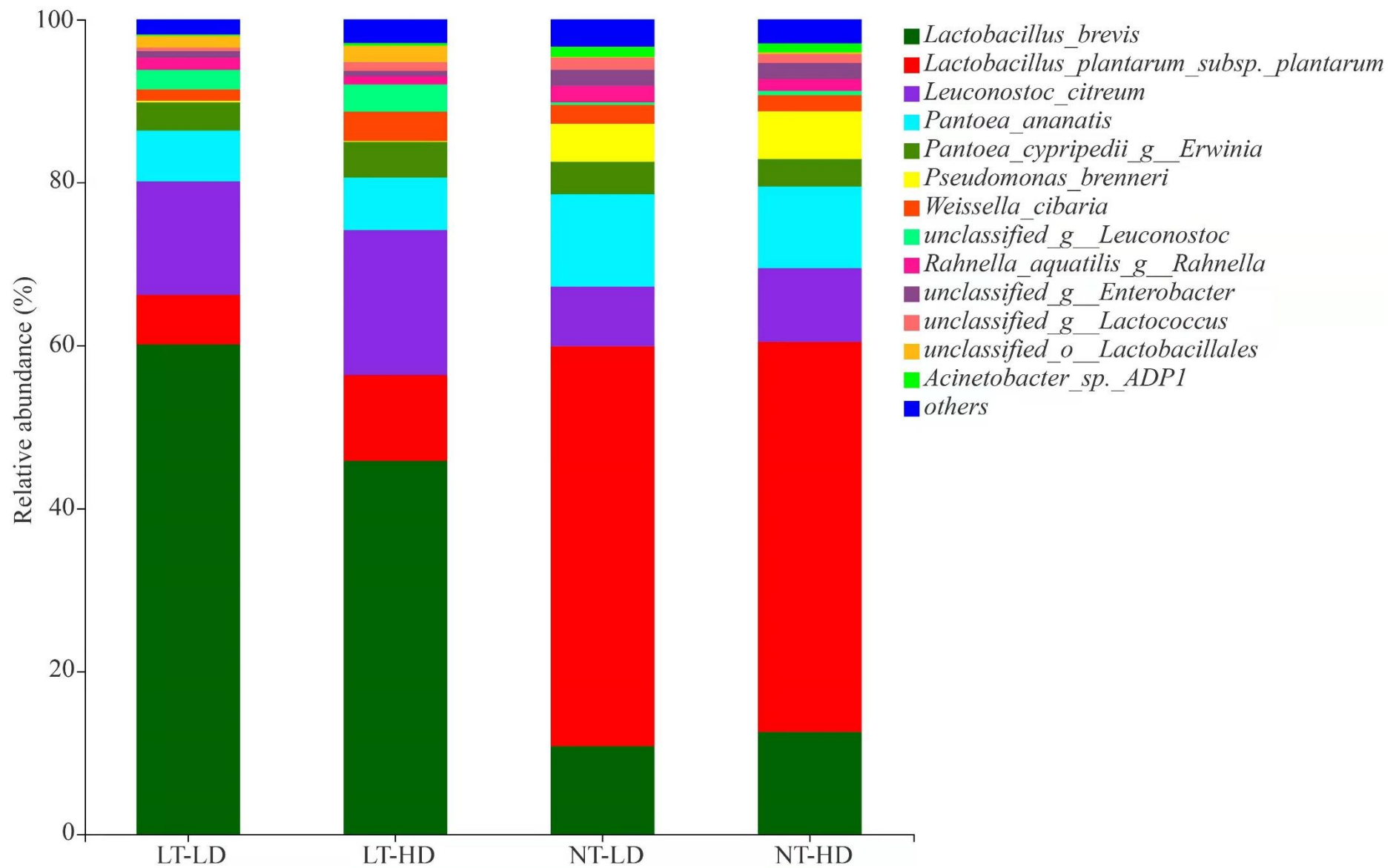

Supplement: Supplementary Figure 1 — Bacteria community (species level) of sorghum-sudangrass silage (n = 3). LT, storage temperature at 10°C; NT, storage temperature at 25°C; LD, ensiling density at 550 kg/m3; HD, ensiling density at 650 kg/m3. [file Data_Sheet_1.PDF]

# Correlation Network

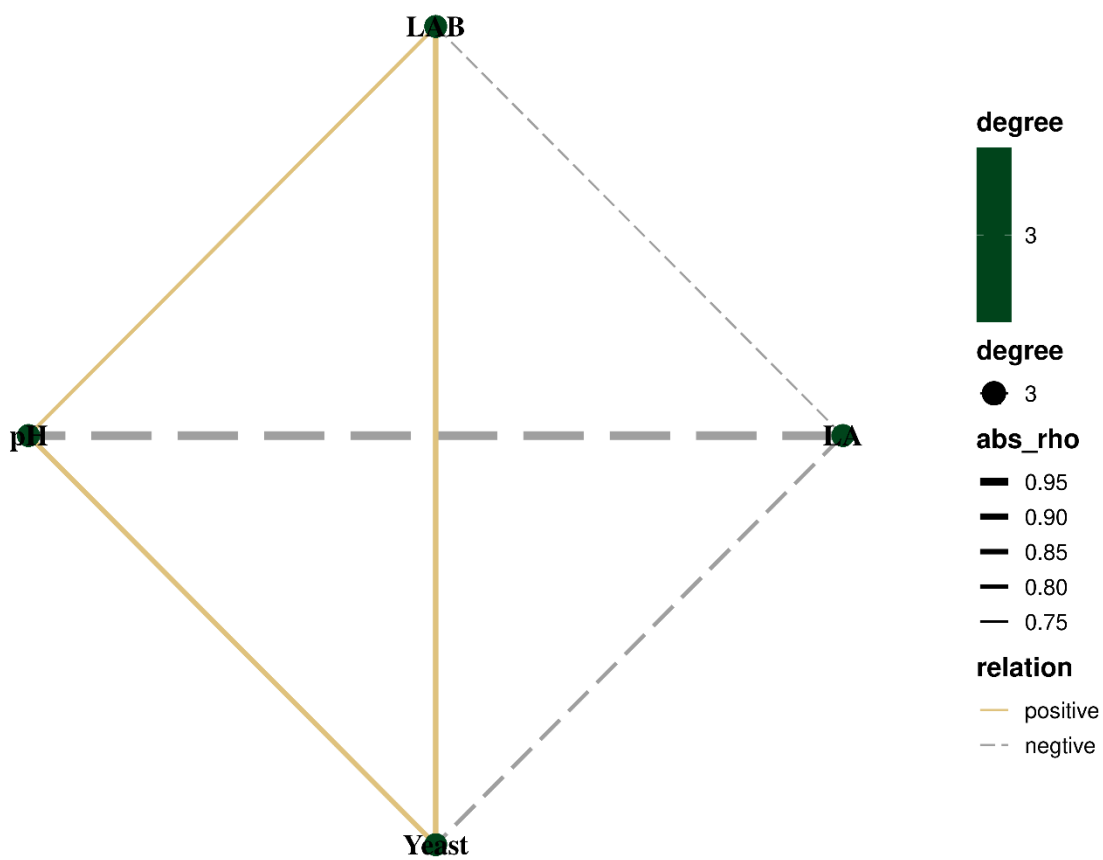

Supplement: Supplementary Figure 2 — Correlation networks among microbial counts and fermentation quality (n = 12). p-Value < 0.05. [file Data_Sheet_2.PDF]
